# Immune Thrombocytopenia Following Booster Dose of the Moderna mRNA-1273 Vaccine

**DOI:** 10.7759/cureus.32873

**Published:** 2022-12-23

**Authors:** Katherine A Breetz, Daniel P Cooper, Shane A Reeves, Samra Haroon Lodhi, Taha Ahmed

**Affiliations:** 1 Internal Medicine, University of Kentucky College of Medicine, Lexington, USA

**Keywords:** covid-19, computed tomography, intravenous immunoglobulin therapy, vaccine-induced thrombocytopenia, immune thrombocytopenic purpura

## Abstract

Vaccine-mediated immune thrombocytopenia, although previously reported, is considered exceedingly rare. The probability of the incidence of profound thrombocytopenia following the COVID-19 mRNA-based vaccine has been less elucidated. We present the case of an 81-year-old female patient who became profoundly thrombocytopenic with bleeding manifestations six days after the Moderna mRNA-1273 vaccine administration. Fortunately, she exhibited platelet count recovery after treatment with intravenous immunoglobulins and steroid therapy. Furthermore, we show that the inherent risk of COVID-19 infection leading to thrombocytopenia significantly outweighs the vaccine’s risk.

## Introduction

Immune thrombocytopenic purpura (ITP) is an autoimmune condition of platelet destruction that results in low platelet counts and a spectrum of clinical presentations ranging from asymptomatic presentations to major bleeding [1]. Vaccine-related ITP has been previously reported for influenza, measles mumps rubella (MMR), and hepatitis vaccines, but such reports have been considered exceedingly rare, and evidence is limited to mostly case reports [1]. However, post-vaccine autoimmune phenomena occur in greater frequency in those who are immunosuppressed due to existing autoimmune diseases and those with a predisposition to its occurrence [2]. Furthermore, in phase III clinical trials for mRNA-based COVID-19 vaccines, thrombocytopenia was rarely noticed, with no significant differences between the vaccinated and placebo groups [3]. An analysis of the Vaccine Adverse Event Reporting System (VAERS) database showed a reporting rate of thrombocytopenia of 0.8 per million mRNA-based COVID-19 vaccine doses. Importantly, most of the cases observed were after the first and second vaccine doses [3]. Herein, we present an intriguing clinical case of an old woman with an uneventful first and second mRNA-based COVID-19 vaccine dose that led to the development of acute ITP related to a booster dose of the Moderna mRNA-1273 vaccine.

## Case presentation

An 81-year-old woman presented to the emergency department of our tertiary care center with worsening epistaxis for four days. She described it as a progressively worsening oozing of blood. One day prior to the presentation, she started noticing large bruising on her arms. Her symptoms were accompanied by fatigue, mild headache, and bilateral leg swelling. Vital signs on initial presentation showed a heart rate of 60 beats per minute, blood pressure of 133/66 mmHg, a temperature of 98.1° F, and a respiratory rate of 16 breaths per minute with 95% saturation on room air. On physical examination, the patient had crusted old blood surrounding the nares, widespread petechiae, multiple bruises along the upper extremities, and petechiae on the chest. She reported compliance with all prescribed medications and only noted her recent changes after receiving the third booster dose of the Moderna mRNA-1273 vaccine 6 days prior to presentation. Initial two doses of vaccination were completed approximately 10 months before the inciting vaccine. Each of her previous vaccines had been tolerated well without any similar symptoms or thrombocytopenia. She denied any history of marked bleeding, recently receiving heparin or blood products, or recent viral or gastrointestinal illness.

The patient had a past medical history of rheumatoid arthritis managed with hydroxychloroquine (initiated three months prior to her presentations), hypertension managed with hydrochlorothiazide, and hypothyroidism managed with levothyroxine.

The initial laboratory tests indicated marked thrombocytopenia (5 × 103/uL), elevated lactate dehydrogenase (261 U/L), and fibrinogen (485 mg/dL) (Table 1).

**Table 1:** Patient's laboratory results at admission. ALT: alanine aminotransferase; APTT: activated partial thromboplastin time; AST: aspartate aminotransferase; LDH: lactate dehydrogenase; PT: prothrombin time; WBC: white blood cell; MCV: mean corpuscular volume.

Laboratory test at admission	Results	Normal value range
Hemoglobin	12 g/dL	11-15.5 g/dL
Platelet count	5 × 10^3^/uL	155-370 × 10^3^/uL
WBC count	3.81 × 10^3^/uL	3.7-10.3 × 10^3^/uL
MCV	88 fL	79-98 fL
Haptoglobin	167 mg/dL	40-220 mg/dL
PT	13.1 seconds	12.2-14.2 seconds
International normalized ratio	1.0	0.9-1.1
Fibrinogen	485 mg/dL	200-460 mg/dL
Creatinine	0.97 mg/dL	0.60-1.10 mg/dL
Urea	17 mg/dL	8-23 mg/dL
Sodium	136 mmol/L	136-145 mmol/L
Potassium	4.8 mmol/L	3.7-4.8 mmol/L
Chloride	102 mmol/L	97-107 mmol/L
Total bilirubin	0.3 mg/dL	0.2-1.1 mg/dL
AST	17 U/L	9-36 U/L
ALT	14 U/L	8-33 U/L
Alkaline phosphatase	62 U/L	46-142 U/L
LDH	261 U/L	116-250 U/L
Albumin	3.6 g/dL	3.5-5.2 g/dL

A peripheral blood smear did not reveal any schistocytes. Given her severe thrombocytopenia, a computed tomography (CT) scan of the head was performed and found to be negative for any acute intracranial abnormality. An ultrasound of the abdomen did not reveal splenomegaly. Reverse transcription PCR testing via nasopharyngeal swab returned negative for COVID-19. Hydroxychloroquine was stopped on admission. Platelet count was 332 × 103/uL two months prior to hospital presentation (Table 2).

**Table 2:** Platelet counts of the patient during the clinical course.

Clinical course	Platelet count (×10^3^/uL)
Two months prior to the presentation	332
Day of admission	5
Post transfusion	37
Day 2	18
Day 3	38
Day 4	62
One-week follow-up	116
Three-month follow-up	134
Six-month follow-up	168

There were no inciting factors for thrombocytopenia in the patient, but she was admitted with a principal diagnosis of vaccine-related ITP. The patient was originally treated with intravenous immunoglobulin (IVIG) 1g/kg, two units of platelets, and intravenous dexamethasone 40 mg. After the second unit of platelets, her count increased to 37 × 103/uL, only to decrease to 18 × 103/uL overnight. The day following the administration of the third unit of platelets, her count increased to 38 × 103/uL, which continued to rise to 62 × 103/uL prior to discharge with two days of IVIG and continued oral prednisone taper.

Since then, she has had regular follow-ups with hematology and rheumatology and exhibited a gradual improvement in platelet count on three- and six-month follow-ups. Given the poor control of rheumatoid arthritis with steroids alone, she was restarted on hydroxychloroquine and did not have another episode of profound thrombocytopenia.

## Discussion

Although uncommon, vaccine-related ITP is not unique to mRNA-based COVID-19 vaccines. Measles, mumps, rubella, and influenza vaccinations have been linked to the development of ITP. The risk of ITP development is further increased in those with a prior history of autoimmune conditions, immunosuppression, or a family predisposition. ITP is not simply a vaccine-related side effect, as approximately 12 per 100,000 adults in the United States develop this condition every year for seemingly unprovoked reasons [4]. With the emergence of COVID vaccines, cases have been presented that indicate a direct association between the new COVID-19 vaccine and the development of ITP. However, data show no direct association between the two, and thrombocytopenia occurs less than expected when the baseline rate of ITP is considered [5]. One report on 20 patients developing ITP related to mRNA-based COVID vaccines showed an onset of symptoms after a median of five days of vaccination, quite similar to our patient [6]. Furthermore, this report indicated a good prognosis with IVIG, steroids, and platelet transfusions, which is also similar to our case [6]. 

The risk of ITP development should not be the determining factor for vaccine compliance, as COVID-19 infection is also associated with many risks, one of them being thrombocytopenia. Up to one-third of COVID-infected patients have thrombocytopenia, of which 7% are asymptomatic and unaware of their COVID-19 infection [7]. Active COVID infection in patients with pre-existing autoimmune conditions has also been suggested as a triggering factor for the relapse of autoimmune conditions [8]. Therefore, the risk of thrombocytopenia in this case report should not be a deterrent to receiving the mRNA-based COVID-19 vaccine, as infection with the virus is more strongly associated with thrombocytopenia. Given that our patient had not developed similar symptoms following the initial vaccination, it can be concluded that thrombocytopenia may occur following any vaccination, the initial or subsequent boosters. 

Considering the proximity of the COVID-19 booster to our patient’s episode of profound thrombocytopenia, her booster was considered the likely inciting factor; however, a multifactorial etiology is likely. Since she had a pre-existing autoimmune condition, as evidence suggests, she may be at an increased risk of relapse as well as post-vaccine autoimmune conditions, including ITP. Although hydroxychloroquine has been linked to thrombocytopenia, the patient’s prolonged course without symptoms and ability to restart medication without any difficulty make it unlikely for hydroxychloroquine to have caused her thrombocytopenia. Additionally, some evidence suggests that hydroxychloroquine may actually be a suitable maintenance treatment for those with ITP [9].

## Conclusions

Although rare and still an evolving topic of research, this case represents a documented case of mRNA vaccine-related ITP secondary to the Moderna mRNA-1273 vaccine for COVID-19. Since this phenomenon is new, this case report helps highlight the prognosis with treatment and follow-up for such a patient. It also indicates the possibility of unique side effects to booster vaccines not witnessed after the initial dose. While, as demonstrated in this paper, there is a risk of thrombocytopenia development in a patient receiving the Moderna mRNA-1273 vaccine, infection with COVID-19 poses an even higher risk. As such, the risk of thrombocytopenia demonstrated in this case report should not be a deterrent to receiving the COVID-19 vaccine, as infection with the virus is more strongly associated with thrombocytopenia development. This case report demonstrates a potential side effect of the mRNA-based COVID-19 vaccine that is important to characterize and understand, especially for patients predisposed to the development of autoimmune conditions. A better understanding of this interaction will allow for a more thorough risk and benefit discussion with individual patients. Such an occurrence should not take away from the benefits of vaccination, and the authors encourage all eligible individuals to receive their vaccinations.

## References

[1] Perricone C, Ceccarelli F, Nesher G (2014). Immune thrombocytopenic purpura (ITP) associated with vaccinations: a review of reported cases. Immunol Res.

[2] Vadalà M, Poddighe D, Laurino C, Palmieri B (2017). Vaccination and autoimmune diseases: is prevention of adverse health effects on the horizon?. EPMA J.

[3] Welsh KJ, Baumblatt J, Chege W, Goud R, Nair N (2021). Thrombocytopenia including immune thrombocytopenia after receipt of mRNA COVID-19 vaccines reported to the Vaccine Adverse Event Reporting System (VAERS). Vaccine.

[4] Idogun PO, Ward MC, Teklie Y, Wiese-Rometsch W, Baker J (2021). Newly diagnosed idiopathic thrombocytopenia post COVID-19 vaccine administration. Cureus.

[5] Iba T, Levy JH (2022). Thrombosis and thrombocytopenia in COVID-19 and after COVID-19 vaccination. Trends Cardiovasc Med.

[6] Lee EJ, Cines DB, Gernsheimer T (2021). Thrombocytopenia following Pfizer and Moderna SARS-CoV-2 vaccination. Am J Hematol.

[7] Bhattacharjee S, Banerjee M (2020). Immune thrombocytopenia secondary to COVID-19: a systematic review. SN Compr Clin Med.

[8] Bobircă A, Bobircă F, Ancuța I (2022). COVID-19-a trigger factor for severe immune-mediated thrombocytopenia in active rheumatoid arthritis. Life (Basel).

[9] Mohammadpour F, Kargar M, Hadjibabaie M (2018). The role of hydroxychloroquine as a steroid-sparing agent in the treatment of immune thrombocytopenia: a review of the literature. J Res Pharm Pract.

